# Outcome of Non-Malignant Papillary Lesions of the Breast on Core Biopsy: An Experience from a Tertiary Care Center in Pakistan

**DOI:** 10.7759/cureus.8364

**Published:** 2020-05-30

**Authors:** Kulsoom Fatima, Shaista Afzal, Muhammad Usman Tariq

**Affiliations:** 1 Radiology, Aga Khan University Hospital, Karachi, PAK; 2 Pathology, Aga Khan University Hospital, Karachi, PAK

**Keywords:** breast papillary lesions, ultrasound, management

## Abstract

Background

Papillary lesions of the breast constitute a heterogeneous group ranging from non-malignant papillomas to papillary carcinoma. While surgical excision is recommended for atypical papilloma or papillary DCIS/ carcinoma on core biopsy, controversy persists in the management of benign papillomas which are diagnosed with core needle biopsy (CNB) since there are variable reported rates for tumor upgrade. The purpose of this study was to determine the outcome of papillary lesions of the breast diagnosed at image-guided CNB, after surgical excision or follow-up, and to identify potential predictors of high-risk lesions/malignancy on imaging.

Materials and methods

We retrospectively identified 52 non-malignant papillary lesions on core biopsy between January 2012 and June 2018. The outcome of surgical excision, as well as clinical and imaging features of these lesions, were assessed. The final histologic upgrade was recorded, and variables were compared between benign and atypical lesions on core biopsy as well as between upgraded and non-upgraded lesions after surgical excision.

Results

Thirty-six lesions out of 52 lesions were benign papillomas on core biopsy, while 16 were papillary lesions with ADH/DCIS. All of these lesions except four benign papillomas were excised. Of the 32 benign papillomas excised, 7 were upgraded to papilloma with ADH/DCIS and one to DCIS with the focus of invasion. Among the 16 atypical lesions excised, one was upgraded to papillary DCIS with a final upgrade rate of 17.3%. There was no statistically significant clinical or imaging feature among those that were upgraded on excision from those that were not upgraded.

Conclusion

Non-malignant papillary lesions have a significant upgrade rate. There are no reliable clinical or imaging features that can pre-surgically predict upgrade. Therefore, surgical excision of all papillary lesions is recommended for definitive diagnosis.

## Introduction

Papillary breast lesions comprise a broad spectrum ranging from benign/atypical papillomas to papillary carcinoma in situ/carcinoma [1]. The reported incidence is up to 6% on core needle biopsy (CNB) specimen [2-4].

The imaging and/or clinical features often pose a diagnostic challenge and cannot reliably predict the benign or malignant nature of the lesion. The initial diagnosis is usually established through a percutaneous biopsy, but the reported false-negative rate is around 3% to 10%. Also, the malignancy rate in papillomas with atypical hyperplasia is about 17% to 67% [5]. The heterogeneity of papillomas as well as under-sampling may account for these observations. While there is consensus that surgical excision is imperative when an atypical papilloma or papillary DCIS/ carcinoma is identiﬁed at CNB, controversy persists in the management of benign papillomas diagnosed at CNB [6]. The published literature supporting their excision reports upgrade rates of 0% to 29% [7]. On the other hand, several authors report either no or minimal upgrade rate and recommend follow-up rather than excision [8]. The decision to evade or pursue surgical excision is crucial as an unwarranted excision leads to potential morbidity and increases healthcare costs. This is especially relevant in our setup as patients pay out of their own pockets and have limited resources. Also, missing a potential malignancy is undesirable as often the patients do not return for follow-up or surveillance due to lack of a proper health system and poor resources.

Therefore, in this study, we intended to determine the outcome of papillary lesions of the breast diagnosed at image-guided CNB after surgical excision or follow-up and compared the ultrasound features of benign and atypical papillary lesions as well as those with the upgrade, thereby identifying the potential predictors of high-risk lesions/malignancy.

## Materials and methods

After approval from the Institutional Ethical Review Committee, the surgical pathology database of the Section of Histopathology, Aga Khan University Hospital was searched for cases reported as "Intra-ductal Papilloma", "Intra-ductal Papilloma with ADH," and "Intra-ductal Papilloma with DCIS" between January 2012 and June 2018, through “Integrated Laboratory Management System (ILMS)” software. 

**Figure 1:**
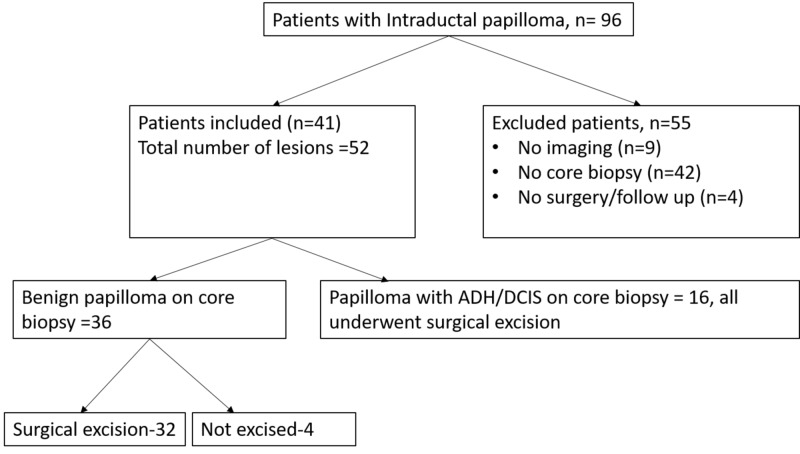
Flow chart showing patients with intraductal papillomas

Cases in which the papillary lesion was identified as an additional finding of invasive carcinoma or cases in which papillary lesion was mixed with other benign proliferative lesions and was difficult to identify as a separate and distinct lesion were excluded. We identified 96 patients (Figure 1). Fifty-five patients were further excluded as they had no prior imaging (n=9), no image-guided core biopsy at our institute (n=42), and had neither surgery nor follow-up imaging available (n=4). Finally, 52 lesions in 41 patients constituted the study population.

All core biopsies were performed under ultrasound guidance with a 14-gauge needle and spring-loaded biopsy gun (Magnum, Bard, Covington, GA) after informed consent. Three to five cores were obtained from each lesion. The core biopsy as well as surgical excision specimens were reviewed by an experienced breast pathologist.

All papillary lesions with ADH or DCIS underwent surgical excision. Benign intra-ductal papillomas either underwent excision (n = 32) or follow-up (n=4) at the discretion of the referring surgeon.

The final histopathology after surgical excision was recorded. The lesions which were benign on initial core biopsy were deemed upgraded if they showed ADH/DCIS on excision. The papillary lesions with ADH or DCIS on initial core biopsy were considered upgraded if they showed papillary DCIS or invasive malignancy after excision.

All statistical analyses were performed using SPSS version 20.0 software. The independent t-test was used to compare continuous variables like age and lesion size, while Chi-square and Fisher’s exact tests were used to compare categorical variables among patients who had benign papillomas with those who had atypia on core biopsy as well as between upgraded and non-upgraded lesions after surgical excision. Two-tailed p-values were calculated. Results with a p-value of less than 0.05 were considered statistically significant.

## Results

A total of 52 non-malignant papillary lesions were found on core biopsy in 41 patients over a six-year period. The mean patient age was 50.8 years (ranging from 21 to 83 years) and the mean lesion size was 13 mm (ranging from 4 to 37 mm).

Seven patients had multiple lesions, while the rest had a solitary lesion.

Thirty-six lesions were classified as benign papillomas while 16 were papillary lesions with ADH/DCIS. All these lesions except four benign papillomas were excised. Three of the benign papillomas that were not excised were followed for 12 months and they showed either decrease in size or disappeared. The fourth one remained unchanged on follow-up (24 months).

The initial pathology at core biopsy and the final pathology at surgical excision are shown in Table 1.

**Table 1:** Histologic findings of non-malignant papillary lesions on core biopsy and surgical excision ADH, atypical ductal hyperplasia; DCIS, ductal carcinoma in situ

Histologic findings at core biopsy	Histologic findings at surgical excision			
	Benign	ADH/DCIS	Papillary DCIS	DCIS with focus of invasion
Benign papillomas that went excision (n = 32)	24	7	0	1
Atypical papillomas (n = 16)	1	14	1	0

Of the 32 benign papillomas excised, seven were found to be atypical (papilloma with ADH/DCIS) and one lesion was upgraded to DCIS with the focus of invasion. Among the 16 atypical lesions excised, one was upgraded to papillary DCIS and one was downgraded to benign papilloma and the rest were unchanged. The final upgrade rate including the four non-excised ones as benign was 17.3%.

Out of 52 papillary lesions, 14 (26.9%) had a palpable mass. Nipple discharge was also present in 14 patients (26.9%), out of which eight had blood discharge. There was a history of either contralateral or ipsilateral malignancy in six patients (11%). On mammography, a mass was detectable in 14 patients (26.9%) and calcification in one patient, while seven patients (13.4%) had either architectural distortion or an indistinct density. In 28 patients, no obvious abnormality was seen on a mammogram due to heterogeneously dense parenchyma. One patient did not undergo a mammogram as she was less than 30 years old and in one patient, a mammogram was not available.

On ultrasound, 29 lesions (55.7%) were intra-ductal, out of which 23 were retro-areolar/central, i.e. within 2 centimeters of the nipple while six of the intra-ductal lesions were peripherally located. The total number of peripherally located lesions was 23 (44.2%) out of the total 52 lesions. Circumscribed margins on ultrasound were seen in 44 (84.6%) patients.

**Figure 2:**
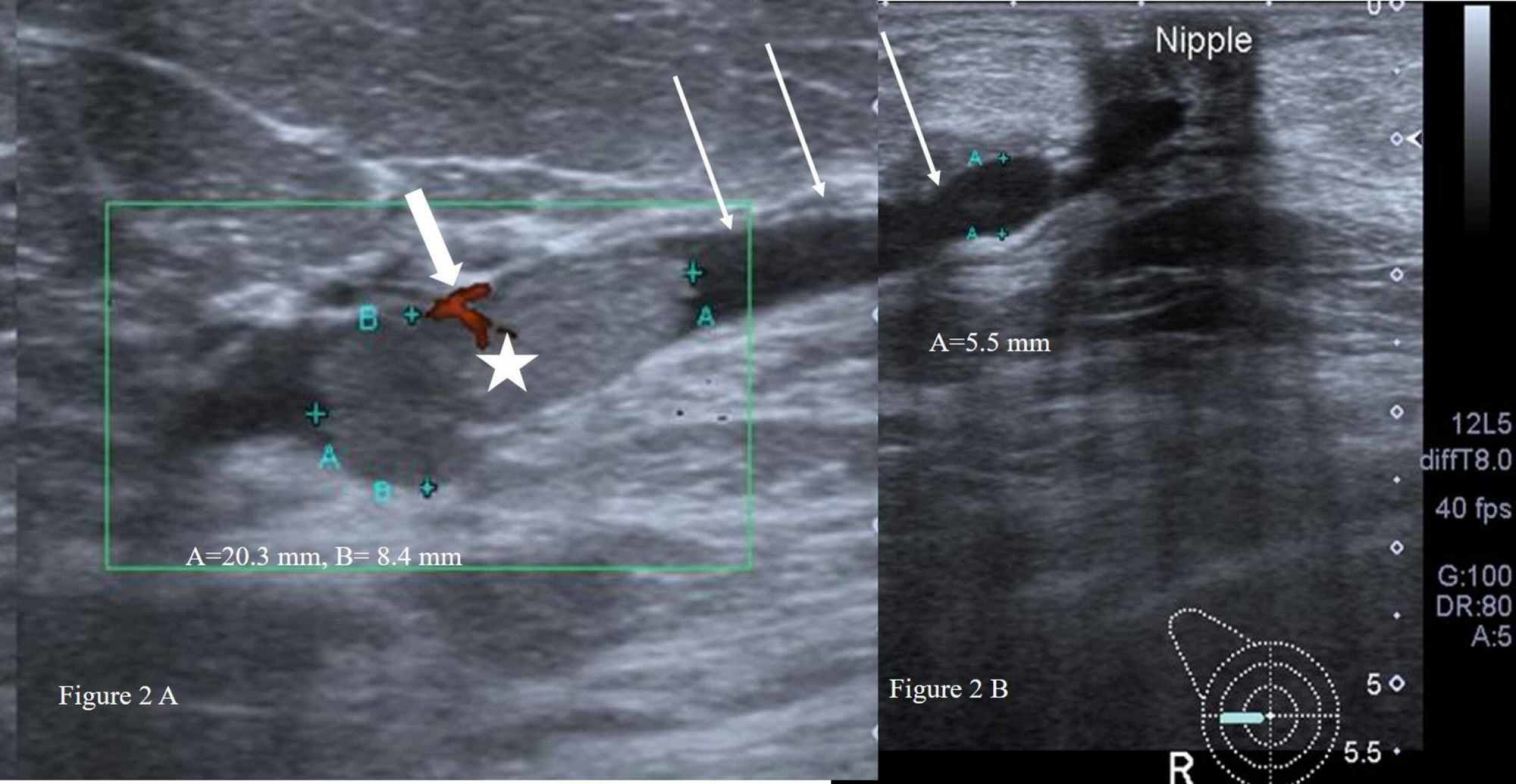
(A) A homogeneous isoechoic solid intraductal nodule (white star) within the dilated duct (long white arrows). Focal vascularity noted within the intraductal nodule (thick short white arrow). (B) The dilated duct (white arrow) is seen extending to the retro-areolar region. The intraductal nodule proved to be benign papilloma on core biopsy as well as surgical excision.

The majority of the lesions with homogeneous echotexture were benign, 31 versus seven atypical lesions (Figure 2). This feature showed a statistical significance with p-value of 0.001. The rest of the clinical and imaging features were not statistically significant among benign and atypical lesions.

The clinical and imaging characteristics of benign and atypical lesions on core biopsy are shown in Table 2.

**Table 2:** Clinical and imaging characteristics of benign and atypical papillary lesions diagnosed on core biopsy

Characteristics	Benign (n=36)	Atypia (n=16)	p-value
Clinical			
Mean age (years)	49.3	49.1	0.96
Mean size (millimeters)	12.5	12.8	0.87
Palpable mass			1.00
Yes	10	04	
No	26	12	
Nipple discharge			0.50
Yes	11	03	
No	25	13	
Contralateral/ ipsilateral malignancy			1.00
Yes	04	02	
No	32	14	
Mammographic findings			
Mass			1.00
Yes	10	04	
No	26	12	
Architectural distortion/indistinct density			0.41
Yes	06	01	
No	30	15	
Ultrasound findings			
Intraductal lesion			0.24
Yes	22	07	
No	14	09	
Vascularity			1.00
Yes	28	13	
No	08	03	
Location			0.96
Central (within 2 cm from nipple)	20	09	
Peripheral	16	07	
Echotexture			0.001
Homogeneous	31	07	
Heterogeneous	05	09	
Margins			0.08
Circumscribed	33	11	
Irregular	03	05	

There was no statistically significant difference in the clinical or imaging features of lesions that were upgraded on excision from those that were not upgraded. Although all upgraded lesions had vascularity on ultrasound, 32 (74.4 %) out of 43 non-upgraded lesions also had vascularity and this feature had an insignificant p value of 0.17. Table 3 summarizes the results of comparison of clinical and imaging features of lesions with histological upgrade to those without upgrade after surgical excision.

**Table 3:** Clinical and imaging characteristics of upgraded and non-upgraded lesions on excision

Characteristics	Upgrade (n=9)	Non- upgrade (n=43)	p-value
Clinical			
Mean age (years)	52.8	48.5	0.33
Mean size (millimeters)	10.5	13	0.30
Palpable mass			0.13
Yes	01	17	
No	08	26	
Nipple discharge			0.68
Yes	03	11	
No	06	32	
Contralateral/ ipsilateral malignancy			1.00
Yes	01	05	
No	08	38	
Mammographic findings			
Mass			1.00
Yes	02	12	
No	07	31	
Architectural distortion/indistinct density			0.59
Yes	02	05	
No	07	38	
Ultrasound findings			
Intraductal lesion			1.00
Yes	05	24	
No	04	19	
Vascularity			0.17
Yes	09	32	
No	00	11	
Location			0.74
Central (within 2 cm from nipple)	06	23	
Peripheral	03	20	
Echotexture			0.41
Homogeneous	08	30	
Heterogeneous	01	13	
Margins			1.00
Circumscribed	08	36	
Irregular	01	07	
Core biopsy result			0.24
Atypia	01	15	
Benign	08	28	

## Discussion

Papillary lesions of the breast are rare, benign papillary lesions representing approximately 10% of breast lesions and invasive papillary carcinoma accounting for only 2% of breast cancer. The diagnosis of papillary lesions is challenging on needle biopsy and sometimes even on excision biopsy, and this has led to controversial and challenging management of these lesions.

Excision has been recommended for papillary lesions with ADH/ALH or atypia as these may be associated with cancer, while for lesions without atypia, the same is not recommended [9]. The underestimation of papillary lesions as reported in a meta-analysis was 15.7% and they found a statistically significant difference of underestimation rate between two groups, i.e. before 2005 vs. 2007 or later (OR 1.974, 95% CI 1.06-3.69) [10]. The improvement in diagnosis was contributed to factors like biopsies under image guidance and immunohistochemistry supporting the pathologist. The upgrade rate in the present study was 17.3%. In a study by Wang et al., the reported upgrade rate of benign /atypical papillary lesions was higher (25%); their study population was primarily African American [11].

The papillary lesions diagnosed on imaging can undergo core biopsy (14 or a 16G needle) or vacuum-assisted Mammotome (MT) biopsy using an 11G needle. It may be difficult to distinguish benign papillary lesion from malignant on core biopsy if only a small part of the lesion is available for pathological review as it depends on the presence or absence of myoepithelial cell layer of the lesion’s papillary component [12]. Hence, insufficient sampling can be one of the factors contributing to the underestimation of papillary lesion especially if a vacuum-assisted larger bore needle is not used. In our study, US-guided 14-G for lesions was performed and may account for the upgrade on subsequent excision.

Due to the reported upgrade rate to cancer, researchers have focused to find the cause of this discrepancy [13-14]. The present study also evaluated a number of demographical and radiological factors for correlation with the upgrade of papillary lesions. The average age of patients with upgraded lesions was 52.8 years which was more than that of patients without upgrading (48.5years), showing results similar to those of Wang et al. although it was not statistically significant [11].

The study by Kil et al. showed two features more frequently with atypical and malignant papillary lesions than benign lesions, i.e. peripheral location and size of more than 1.5 cm; these features were not seen in the present study [1]. The size of the papilloma was also evaluated for the likelihood of malignancy by Glenn et al.; however, no correlation with the size of the lesion was seen in their study [15]. Hence, the size threshold for surgical excision cannot be recommended. 

Although great efforts have been made to determine the predictive value of a number of clinical and imaging features for up-gradation of papillary lesions on excisional biopsy, the conclusions from these studies vary significantly [16-18]. In the present study, none of the clinical features showed statistical significance in predicting the upgrade of the lesion.

Small retro areolar papillomas are often difficult to identify on mammography. Larger lesions may appear as circumscribed round or oval masses with or without calcification [19-20]. In our study, more than half of the lesions (53.8%) were not discernable on mammograms due to heterogeneously dense parenchyma. The mammographic features observed in the remaining cases were either mass or an indistinct density. Calcification was a very rare feature, seen only in one case. None of the mammographic features showed statistical significance for predicting atypia or upgrade.

The typical ultrasound features of a benign intraductal papilloma are a solid nodule within a dilated duct in the subareolar location [21]. In the present study, out of total 28 non-upgraded benign papillomas, 13 were peripherally located, among which seven were not intraductal. Also, five of the upgraded benign papillomas were retro areolar as well as intraductal. Similarly, more than half of the atypical papillomas (56.2%), including the ones with upgrades were retro areolar. Hence, this feature too cannot reliably differentiate benign from atypical papilloma.

The other sonographic features of papillary breast lesions suspicious for malignancy include irregular, non-circumscribed mass, non-parallel to the skin, complex echo pattern, posterior acoustic shadowing, or a combination of features [22]. In the present study, although the majority of benign papillomas were circumscribed, the p-value was not statistically significant (0.08). Another significant finding, i.e. posterior acoustic shadowing, was not observed in any lesion whether benign, atypical, or malignant. The only sonographic feature which showed a statistical significance (p-value, 0.001) among benign and atypical papillomas was echotexture. Most of the lesions with homogeneous echotexture were benign. However, the implication of this is uncertain as all eight benign papillomas that were finally upgraded also had homogeneous echotexture. We, therefore, recommend surgical excision of all non-malignant papillary lesions.

This study has a few limitations; first, this is a single-center study; second, this has a small sample size due to the rarity of papillary lesions of the breast. Further multicenter studies are thus required for the determination of clinical and imaging risk factors associated with the underestimation of these lesions.

## Conclusions

Non-malignant papillary breast lesions show significant upgrade on excision whether they are benign papillomas or those with atypia. There are no reliable clinical or imaging features that can predict the upgrade or accurately differentiate benign from atypical papillary lesions pre-surgery. The inadequate sampling on core biopsy is an unavoidable persistent issue and continues to account for underestimation. We therefore recommend surgical excision of all papillary lesions diagnosed on core biopsy for definitive evaluation.

## References

[1] Kil WH, Cho EY, Kim JH, Nam SJ, Yang JH (2008). Is surgical excision necessary in benign papillary lesions initially diagnosed at core biopsy?. Breast.

[2] Liberman L, Bracero N, Vuolo MA, Dershaw DD, Morris EA, Abramson AF, Rosen PP (1999). Percutaneous large-core biopsy of papillary breast lesions. AJR Am J Roentgenol.

[3] Mercado CL, Hamele-Bena D, Singer C, Koenigsberg T, Pile-Spellman E, Higgins H, Smith SJ (2001). Papillary lesions of the breast: evaluation with stereotactic directional vacuum-assisted biopsy. Radiology.

[4] Kim MJ, Kim SI, Youk JH, Moon HJ, Kwak JY, Park BW, Kim EK (2011). The diagnosis of non-malignant papillary lesions of the breast: comparison of ultrasound-guided automated gun biopsy and vacuum-assisted removal. Clin Radiol.

[5] Jackman RJ, Nowels KW, Rodriguez-Soto J, Marzoni Jr FA, Finkelstein SI, Shepard MJ (1999). Stereotactic, automated, large-core needle biopsy of nonpalpable breast lesions: false-negative and histologic underestimation rates after long-term follow-up. Radiology.

[6] Cyr AE, Novack D, Trinkaus K (2011). Are we overtreating papillomas diagnosed on core needle biopsy?. Ann Surg Oncol.

[7] Destounis S, Seifert P, Somerville P, Murphy P, Morgan R, Arieno A, Young WL (2014). Underestimation of papillary breast lesions by core biopsy: correlation to surgical excision. Breast Cancer.

[8] Grimm LJ, Bookhout CE, Bentley RC, Jordan SG, Lawton TJ (2018). Concordant, non-atypical breast papillomas do not require surgical excision: a 10-year multi-institution study and review of the literature. Clin Imaging.

[9] Menes TS, Rosenberg R, Balch S, Jaffer S, Kerlikowske K, Miglioretti DL (2014). Upgrade of high-risk breast lesions detected on mammography in the Breast Cancer Surveillance Consortium. Am J Surg.

[10] Wen X, Cheng W (2013). Nonmalignant breast papillary lesions at core-needle biopsy: a meta-analysis of underestimation and influencing factors. Ann Surg Oncol.

[11] Wang H, Tsang P, D’Cruz C, Clarke K (2014). Follow-up of breast papillary lesion on core needle biopsy: experience in African-American population. Diagn Pathol.

[12] Han BK, Choe YH, Ko YH, Yang JH, Nam SJ (1999). Benign papillary lesions of the breast: sonographic‐pathologic correlation. J Ultrasound Med.

[13] Menes TS, Rosenberg R, Balch S, Jaffer S, Kerlikowske K, Miglioretti DL (2014). Upgrade of high-risk breast lesions detected on mammography in the Breast Cancer Surveillance Consortium. Am J Surg.

[14] Shiino S, Tsuda H, Yoshida M, Jimbo K, Asaga S, Hojo T, Kinoshita T (2015). Intraductal papillomas on core biopsy can be upgraded to malignancy on subsequent excisional biopsy regardless of the presence of atypical features. Pathol Int.

[15] Glenn ME, Throckmorton AD, Thomison JB, Bienkowski RS (2015). Papillomas of the breast 15 mm or smaller: 4-year experience in a community-based dedicated breast imaging clinic. Ann Surg Oncol.

[16] Hong ZJ, Chu CH, Fan HL, Hsu HM, Chen CJ, Chan DC, Yu JC (2011). Factors predictive of breast cancer in open biopsy in cases with atypical ductal hyperplasia diagnosed by ultrasound-guided core needle biopsy. Eur J Surg Oncol.

[17] Tokiniwa H, Horiguchi J, Takata D (2011). Papillary lesions of the breast diagnosed using core needle biopsies. Exp Ther Med.

[18] Wiratkapun C, Keeratitragoon T, Lertsithichai P, Chanplakorn N (2013). Upgrading rate of papillary breast lesions diagnosed by core-needle biopsy. Diagn Interv Radiol.

[19] Eiada R, Chong J, Kulkarni S, Goldberg F, Muradali D (2012). Papillary lesions of the breast: MRI, ultrasound, and mammographic appearances. AJR Am J Roentgenol.

[20] Woods ER, Helvie MA, Ikeda DM, Mandell SH, Chapel KL, Adler DD (1992). Solitary breast papilloma: comparison of mammographic, galactographic, and pathologic findings. AJR Am J Roentgenol.

[21] Kim TH, Kang DK, Kim SY, Lee EJ, Jung YS, Yim H (2008). Sonographic differentiation of benign and malignant papillary lesions of the breast. J Ultrasound Med.

[22] Lam WW, Chu WC, Tang AP, Tse G, Ma TK (2006). Role of radiologic features in the management of papillary lesions of the breast. AJR Am J Roentgenol.

